# Microencapsulation: A promising technique for controlled drug delivery

**Published:** 2010

**Authors:** M.N. Singh, K.S.Y. Hemant, M. Ram, H.G. Shivakumar

**Affiliations:** Department of Pharmaceutics, JSS College of Pharmacy, SS Nagar, Mysore, Karnataka-570015, India

**Keywords:** Drug delivery systems, Microcapsules, Controlled release, Microencapsulation

## Abstract

Microparticles offer various significant advantages as drug delivery systems, including: (i) an effective protection of the encapsulated active agent against (e.g. enzymatic) degradation, (ii) the possibility to accurately control the release rate of the incorporated drug over periods of hours to months, (iii) an easy administration (compared to alternative parenteral controlled release dosage forms, such as macro-sized implants), and (iv) Desired, pre-programmed drug release profiles can be provided which match the therapeutic needs of the patient. This article gives an overview on the general aspects and recent advances in drug-loaded microparticles to improve the efficiency of various medical treatments. An appropriately designed controlled release drug delivery system can be a foot ahead towards solving problems concerning to the targeting of drug to a specific organ or tissue, and controlling the rate of drug delivery to the target site. The development of oral controlled release systems has been a challenge to formulation scientist due to their inability to restrain and localize the system at targeted areas of gastrointestinal tract. Microparticulate drug delivery systems are an interesting and promising option when developing an oral controlled release system. The objective of this paper is to take a closer look at microparticles as drug delivery devices for increasing efficiency of drug delivery, improving the release profile and drug targeting. In order to appreciate the application possibilities of microcapsules in drug delivery, some fundamental aspects are briefly reviewed.

## INTRODUCTION

Controlled drug delivery technology represents one of the frontier areas of science, which involves multidisciplinary scientific approach, contributing to human health care. These delivery systems offer numerous advantages compared to conventional dosage forms, which include improved efficacy, reduced toxicity, and improved patient compliance and convenience. Such systems often use macromolecules as carriers for the drugs. By doing so, the treatments that would not otherwise be possible are now in conventional use. This field of pharmaceutical technology has grown and diversified rapidly in recent years. Understanding the derivation of the methods of controlled release and the range of new polymers can be a barrier to involvement of the non-specialist. Of the different dosage forms reported, nanoparticles and microparticles attained much importance, due to a tendency to accumulate in inflamed areas of the body. Nano and microparticles for their attractive properties occupy unique position in drug delivery technology. Some of the current trends in this area will be discussed(1–3).

The terminology used to describe micro-particulate formulations can sometimes be inconsistent and confusing to readers unfamiliar with the field. Basically, the term “microparticle” refers to a particle with a diameter of 1-1000 μm, irrespective of the precise interior or exterior structure. Within the broad category of microparticles, “microspheres” specifically refers to spherical microparticles and the subcategory of “micro-capsules” applies to microparticles which have a core surrounded by a material which is distinctly different from that of the core. The core may be solid, liquid, or even gas(4–6)

**Fig. 1:**
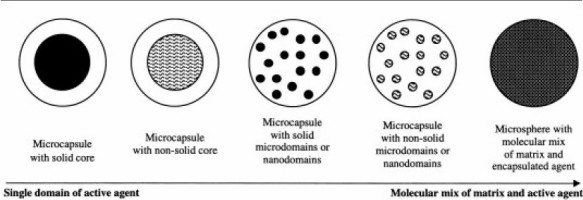
Different structures of microcapsules and microsphere (1).

Despite the specific and logical subcategories, many researchers use the terms interchangeably, which often leads to the confusion of the reader. It is usually assumed that a formulation described as a microsphere is comprised of a fairly homogeneous mixture of polymer and active agent, whereas microcapsules have at least one discrete domain of active agent and sometimes more. Some variations on microparticle structures are given in Fig. 1. As the domains and subdomains of active agent within micro-capsules become progressively smaller, the microcapsules become microparticles(7–9)

The term “microcapsule” is defined, as a spherical particle with the size varying between 50 nm to 2 mm containing a core substance. Microspheres are in strict sense, spherically empty particles. However, the terms microcapsules and microspheres are often used synonymously. In addition, some related terms are used as well. For example, “microbeads” and “beads” are used alternatively. Sphere and spherical particles are also employed for a large size and rigid morphology. Due to attractive properties and wider applications of microcapsules and microspheres, a survey of the applications in controlled drug release formulations is appropriate(1,6,7).

Although the word capsule implies a core and shell structure, the term microcapsules admits not only membrane enclosed particles or droplets but also dispersion in solid matrix lacking a distinctive external wall phase as well as intermediate types. The size range (2 to 2000 μm approximately) distinguishes them from the smaller nanoparticles or nanocapsules.

**Fig. 2:**
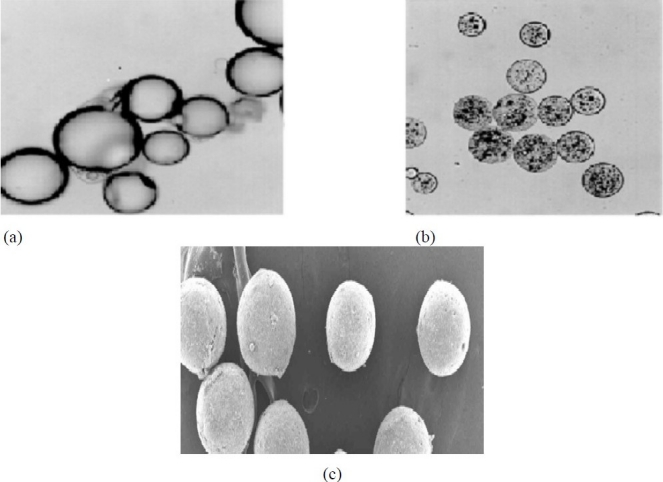
a) Mononuclear microcapsules, b) Aggregated microcapsules, c) Surface of microcapsule (11).

The scanning electron microscopy (SEM) has revealed the structural features of microcapsules as to be varying and complex. The walled prototype may be mononuclear as shown in Fig. 2a, or may have multiple core structure. Also double or multiple concentric coating may be present. Aggregated microcapsules greatly vary in size and shape (Fig. 2b), and may also posses additional external wall. The perfect microcapsules are obtainable by using the liquid cores or forming the microcapsules as a liquid dispersed phase prior to the solidification. Although micro-structure of both membrane and interior can be detected by SEM of surfaces or sections (Fig. 2c), their physical quality, involving porosity, tortuousity and crystallinity, is difficult to be characterized quantitatively in microcapsules. However, some progress has been made, and efforts are continuing to calculate permeability and porosity from release data, dimensions, densities, and core/wall ratios. The effect of size and shape distribution has only been studied recently(8–10)

Microcapsules are finally dispersed in various dosage forms, such as hard gelatin capsules, which may be enteric coated, soft gelatin capsules, or suspensions in liquids, all of which allow dispersion of individual microcapsules on release.

Microcapsules continue to be of much interest in controlled release because of relative ease in design and formulation and partly on the advantages of microparticulate delivery systems. The latter include sustained release from each individual microcapsule and offer greater uniformity and reproducibility. Additional advantage over monolithic systems containing multiple doses is the greater safety factor in case of a burst or defective individual in subdivided dosage forms. Finally, it has been argued that multiple particle systems are distributed over a great length of gastro-intestinal tract, which should result in, (a) lowered local concentrations and hence reduced toxicity or irritancy, and (b) reduced variability in transit time and absorption rate(12–14)

### Composition of microcapsules

#### Coating materials

A wide variety of coating materials are available for microencapsulation. Some patent innovative coating polymers have also been developed for some special applications particularly among the bioadhesives and mucoadhesives. However, many traditional coating materials are satisfactory for the use in the gastrointestinal tract. They include inert polymers and pH sensitive ones as carboxylate and amino derivatives, which swell or dissolve according to the degree of cross-linking(15–19)

The selection of appropriate coating material from a long list of candidate materials needs consideration of the following general criteria by the research pharmacist:

1. What are the specific dosage forms or product requirements, such as stabilization, reduced volatility, release characteristics, and environmental conditions?
2. What coating material will satisfy the product objective and requirements?
3. What microencapsulation method is best suited to accomplish the coated product objectives?

The selection of appropriate coating material decides the physical and chemical properties of the resultant microcapsules/ microspheres. While selecting a polymer the product requirements i.e. stabilization, reduced volatility, release characteristics, environmen-tal conditions, etc. should be taken into consideration. The polymer should be capable of forming a film that is cohesive with the core material. It should be chemically compatible, non-reactive with the core material and provide the desired coating properties such as strength, flexibility, impermeability, optical properties and stability(1,5,20–22)

Generally hydrophilic polymers, hydrophobic polymers or a combination of both are used for the microencapsulation process. A number of coating materials have been used successfully; examples of these include gelatin, polyvinyl alcohol, ethyl cellulose, cellulose acetate phthalate and styrene maleic anhydride. The film thickness can be varied considerably depending on the surface area of the material to be coated and other physical characteristics of the system. The micro-capsules may consist of a single particle or clusters of particles. After isolation from the liquid manufacturing vehicle and drying, the material appears as a free flowing powder. The powder is suitable for formulation as compressed tablets, hard gelatin capsules, suspensions, and other dosage forms(23,26)

#### Core materials

The core material is the material over which coating has to be applied to serve the specific purpose. Core material may be in form of solids or droplets of liquids and dispersions. The composition of core material can vary and thus furnish definite flexibility and allow effectual design and development of the desired microcapsule properties. A substance may be microencapsulated for a number of reasons. Examples may include protection of reactive material from their environment, safe and convenient handling of the materials which are otherwise toxic or noxious, taste masking, means for controlled or modified release properties means of handling liquids as solids, preparation of free flow powders and in modification of physical properties of the drug(5,27–32)

### Technologies used for the preparation of microcapsules

**Fig. 3:**
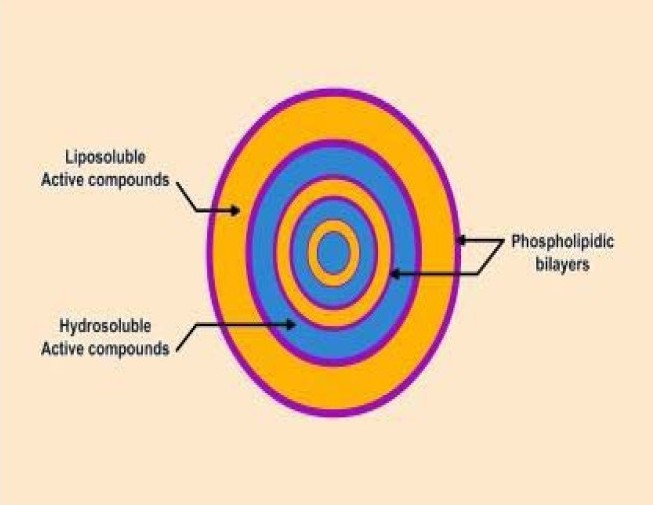
The microencapsulation process (41).

The method of preparation and the techniques employed for microencapsulation overlap considerably (Fig. 3). The various microencapsulation processes can be divided into chemical, physiochemical, and electrostatic and mechanical processes. Chemical processes include the interfacial and *in situ* polymerization methods. Physiochemical processes include coacervationphase separation, complex emulsion, meltable dispersion and powder bed methods. Mechanical processes include the air-suspension method, pan coating, and spray drying, spray congealing, micro-orifice system and rotary fluidization bed granulator method. Also the spheronization is some times included under the mechanical process of microencapsulation. Sustained release polymers microcapsules containing drug with various solubility characteristics were prepared with colloidal polymer dispersion in a completely aqueous environment as an alternative to the conventional microencapsulation technique(5,33,40)

The microencapsulation by coacervation-phase separation generally consists of three steps carried out under continuous agitation: (a) formation of three immiscible chemical phases, (b) deposition of coating, and (c) rigidization of the coating. The coacervation-phase separation has been classified into two categories, simple coacervation and complex coacervation. The former implies addition of a strongly hydrophilic substance to a solution of colloid. This added substance causes two phases to be formed. The complex coacer-vation is principally a pH dependant process. The acidic or basic nature of the system is manipulated to produce microcapsules. Above a certain critical pH value, the system depending upon its acidic or basic nature may produce microcapsules. Below that pH value they will not be formed. Usually complex coacervation deals with the system containing more than one colloid(42–44)

In interfacial polymerization, a monomer is made to be polymerized at the interface of two immiscible substances. If the internal phase is a liquid, it is possible to disperse or solublize the monomer in this phase and emulsify the mixture in the external phase until the desired particle size is reached. At this point a cross-linking agent may be added to the external phase. Since there is usually some migration of the monomer from the internal to external phase, and since it is preferred that the cross-linking agent does not transfer to the internal phase, the bulk of any polymerization will take place at the interface(45–47).

The electrostatic methods of microencapsulation involve trigging together the wall material and the material to be encapsulated when both are aerosolized. The wall material must be liquid during encapsulation stage and must be capable of surrounding the core material. The aerosols produced must be oppositely charged. Three chambers are used for the process, two for atomization of the wall and core material and the third for mixing. Oppositely charged ions are generated and deposited on the liquid drops while they are atomized(1,6,21,48)

Mechanical methods used for microencapsulation utilize the special equipments for their own. The microcapsules produced result from mechanical procedures rather than from a well-defined physical or chemical phenomenon. The most commonly employed mechanical methods for the preparation of micro-capsules and microspheres are(1,5,48,49):

(a) Multiorifice-centrifugal process, developed by the Southwest Research Institute as a mechanical process for producing micro-capsules that utilizes centrifugal forces to hurl a core material particle trough an enveloping microencapsulation membrane thereby affecting mechanical microencapsulation. The multiorifice-centrifugal process is capable for microencapsulating liquids and solids of varied size ranges, with diverse coating materials.

(b) Air suspension coating (wurster) consist of the dispersing of solid, particulate core materials in a supporting air stream and the spray coating on the air suspended particles.

(c) Spray drying and Spray congealing, both methods have been used for many years as microencapsulation techniques. Because of certain similarities of the two processes, they are discussed together. Spray drying and spray congealing processes are similar in that both involve dispersing the core material in a liquefied coating substance and spraying or introducing the core coating mixture into some environmental condition, whereby relatively rapid solidification of the coating is affected. The principal difference between the two methods is coating solidification. Coating solidification in the case of spray drying is affected by rapid evaporation of a solvent in which the coating material is dissolved whereas in spray congealing it is accomplished by thermally congealing a molten coating material or by solidifying a dissolved coating by introducing the coating core material mixture into a nonsolvent.

(d) Pan coating, for the microencapsulation of relatively large particles, has become wide spread in the pharmaceutical industry. With respect to microencapsulation, solid particles greater than 600 microns in size are generally considered essential for effective coating and the process has been extensively employed for the preparation of controlled release beads. In practice, the coating is applied as a solution or as an atomized spray to the desired solid core material in the coating pan. Usually, to remove the coating solvent, warm air is passed over the coated materials as the coatings are being applied in the coating pans.

### Mechanism and kinetics of drug release

Major mechanisms of drug release from microcapsules include diffusion, dissolution, osmosis and erosion.

#### Diffusion

Diffusion is the most commonly involved mechanism wherein the dissolution fluid penetrates the shell, dissolves the core and leak out through the interstitial channels or pores. Thus, the overall release depends on, (a) the rate at which dissolution fluid penetrates the wall of microcapsules, (b) the rate at which drug dissolves in the dissolution fluid, and (c) the rate at which the dissolved drug leak out and disperse from the surface(3,4,16). The kinetics of such drug release obeys Higuchi’s equation as below(4,5,8,50,51):

$$Q\;=\;{\left[D/J\;\left(2A\;-\;\varepsilon \;C_{S}\right)\;C_{S}\;t\right]}^{1/2}$$

Where, Q is the amount of drug released per unit area of exposed surface in time t; D is the diffusion coefficient of the solute in the solution; A is the total amount of drug per unit volume; C_S_ is the solubility of drug in permeating dissolution fluid; ε is the porosity of the wall of microcapsule; J is the tortuosity of the capillary system in the wall. The above equation can be simplified to Q = vt where, v is the apparent release rate.

#### Dissolution

Dissolution rate of polymer coat determines the release rate of drug from the microcapsule when the coat is soluble in the dissolution fluid. Thickness of coat and its solubility in the dissolution fluid influence the release rate(5,6,52)

#### Osmosis

The polymer coat of microcapsule acts as semi permeable membrane and allows the creation of an osmotic pressure difference between the inside and the outside of the microcapsule and drives drug solution out of the microcapsule through small pores in the coat(7,53)

#### Erosion

Erosion of coat due to pH and/or enzymatic hydrolysis causes drug release with certain coat materials like glyceryl monostearate, bee’s wax and stearyl alcohol(13,54)

Attempts to model drug release from microcapsules have become complicated due to great diversity in physical forms of microcapsules with regard to size, shape and arrangement of the core and coat materials(1,4,6,55). The physiochemical properties of core materials such as solubility, diffusibility and partition coefficient, and of coating materials such as variable thickness, porosity, and inertness also makes modeling of drug release difficult. However, based on various studies concerning the release characteristics, the following generalizations can be made:

1. Drug release rate from microcapsules conforming to reservoir type is of zero order.
2. Microcapsules of monolithic type and containing dissolved drug have release rates that are t_1/2_ dependant for the first half of the total drug release and thereafter decline exponentially.
3. However, if a monolithic microcapsule containing large excess of dissolved drug, the release rate is essentially t_1/2_ dependant throughout almost the entire drug release.

In monolithic capsules the path traveled by drug is not constant; the drug at the center travels a large distance than the drug at the surface. Therefore, the release rate generally decreases with time.

### Applications of microcapsules and microspheres

Some of the applications of microencapsulation can be described in detail as given below:

1. Prolonged release dosage forms. The microencapsulated drug can be administered, as microencapsulation is perhaps most useful for the preparation of tablets, capsules or parenteral dosage forms(3)
2. Microencapsulation can be used to prepare enteric-coated dosage forms, so that the medicament will be selectively absorbed in the intestine rather than the stomach(56)
3. It can be used to mask the taste of bitter drugs(6,57)
4. From the mechanical point of view, microencapsulation has been used to aid in the addition of oily medicines to tableted dosage forms. This has been used to overcome problems inherent in producing tablets from otherwise tacky granulations and in direct compression to tablets(58,59)
5. It has been used to protect drugs from environmental hazards such as humidity, light, oxygen or heat. Microencapsulation does not yet provide a perfect barrier for materials, which degrade in the presence of oxygen, moisture or heat, however a great degree of protection against these elements can be provided(60,61)
6. The separations of incompatible substances, for example, pharmaceutical eutectics have been achieved by encapsulation. This is a case where direct contact of materials brings about liquid formation. The stability enhancement of incompatible aspirin-chlorpheniramine maleate mixture was accomplished by micro-encapsulating both of them before mixing(6)
7. Microencapsulation can be used to decrease the volatility. An encapsulated volatile substance can be stored for longer times without substantial evaporation(6)
8. Microencapsulation has also been used to decrease potential danger of handling of toxic or noxious substances. The toxicity occurred due to handling of fumigants, herbicides, insecticides and pesticides have been advantageously decreased after microencapsulation(61)
9. The hygroscopic properties of many core materials may be reduced by microencapsulation(62)
10. Many drugs have been microencapsulated to reduce gastric irritation(62)
11. Microencapsulation method has also been proposed to prepare intrauterine contraceptive device(12,63)
12. In the fabrication of multilayered tablet formulations for controlled release of medi-cament contained in medial layers of tableted particles(1,7,10,63)

### Recent advances in microencapsulation

Several methods and techniques are potentially useful for the preparation of polymeric microparticles in the broad field of microencapsulation. The preparation method determines the type and the size of microparticle and influence the ability of the interaction among the components used in microparticle formulations. The term microparticle designates systems larger than one micrometer in diameter and is used usually to describe both microcapsules and microspheres. Microparticles-containing drugs are employed for various purposes including -but not restricted to- controlled drug delivery, masking the taste and odor of drugs, protection of the drugs from degradation, and protection of the body from the toxic effects of the drugs. Polymeric carriers being essentially multi-disciplinary are commonly utilized in microparticle fabrication and they can be of an erodible or a non-erodible type(63)

Recently, numbers of publications and patents have been published. Hughes(64) provided a method of sustained delivery of an active drug to a posterior part of an eye of a mammal to treat or prevent a disease or condition affecting mammals. The method is comprised of administering an effective amount of an ester prodrug of the active drug such as tazarotene (prodrug of tazarotenic acid) subconjunctivally or periocularly since a systemic administration requires high systemic concentration of the prodrug. The ester prodrug is contained in biodegradable polymeric microparticle system prepared using the o/w emulsion solvent evaporation methods. Lee et al.(65,66) prepared a composition in the form of thin film or strip composed of microspheres containing antibiotic such as minocycline HCl. It was made using a biodegradable polymer, prepared by a modified o/w emulsification technique followed by solvent evaporation. Water-soluble polysaccharide polymers such as pectin was used for making thin film or strip containing microspheres intended for local sustained release administration into the periodontal pocket. The thin film or strip is coated by spray-coating with cation salt aqueous solution of calcium or barium chlorides. In one embodiment, Traynor et al. used the o/w emulsion to produce sol-gel microcapsules (containing sunscreens) that are highly positively charged using non-ionizing cationic additives which can include cationic polymers(67)

An injectable slow-release partial opioid agonist or opioid antagonist in a poly (D, L-lactide) microspheres with a small amount of residual ethyl acetate was provided by Tice et al.(68) and Markland et al.(69) where an o/w emulsion is first prepared from an organic phase made of ethyl acetate and an aqueous phase comprised an aqueous ethyl acetate containing solution of polyvinyl alcohol. Microspheres are recovered by extraction with water. Wen and Anderson(70) prepared single wall biodegradable microspheres by extracting an o/w emulsion containing steroidal and non-steroidal anti-inflammatory agents. Otherwise, double wall microspheres were prepared. Microspheres containing the active ingredient were then immobilized on a substrate surface in a polymeric matrix that is an implantable medical article or an *in situ* formed matrix. Solidification method of the hydrophilic capsule materials such as gelatin can be through rapidly lowering the temperature and subsequent dehydration. While such method achieved some significant commercial success, difficulties have sometimes been encountered in rapidly inducing solidification of the microencapsulating material.

The use of various gel forming proteins (collagen and gelatin) and polysaccharides (agar, calcium alginate, and carrageenan) introduced a milder, biocompatable immobilization or isolation system. Obeidat and Price(71) employed a one step method for the preparation of microspheres having enteric and controlled release characteristics in one embodiment and swelling and controlled properties in an other using the nonaqueous solvent evaporation method. Microspheres were especially useful for delivery of moderately non-polar active ingredients but can be formulated to deliver very soluble polar compounds.

Delgado(72) developed a method for preparing enteric polymeric microparticles containing a proteinaceous antigen in a single or double emulsification process in which the enteric polymer acts as a stabilizer for the microparticles which are formed in the process.

Single o/w or double w/o/w emulsion solvent evaporation method was utilized by Yamamoto et al.(73–75)to prepare microspheres with improved dispersibility by dispersing a w/o type emulsion in an outer aqueous phase that contains an osmotic pressure regulating agent(73) or to prepare sustained release microsphere containing a LHRH derivative or its salt in a large amount without containing gelatin by using a lactic acid-glycolic acid polymer or salts. When the low molecular weight of lactic acid-glycolic acid polymer fraction (8,000 to about 15,000) is contained in a large amount, LHRH derivative readily interacts with these polymers of high reactivity(74) or otherwise to produce a sustained-release composition which comprises emulsifying an aqueous solution containing LHRH derivative and an acid or a base with a solution of a biodegradable polymer(75)

Rickey et al.(76) provided a novel method for the preparation of biodegradable and biocompatible microparticles containing a biologically active agent such as risperidone, or testosterone dissolved in a blend of at least two substantially non-toxic solvents, free of halogenated hydrocarbons such as benzyl alcohol and ethyl acetate. The blend was dispersed in an aqueous solution to form droplets. The resulting emulsion was then added to an aqueous extraction medium. One of the solvents in the solvent blend would be extracted in the quench step (aqueous solution) more quickly than the other solvent. Owing to the high boiling point of the left solvent (benzyl alcohol) which is not easily removed by evaporation in air or other conventional evaporative means, some of the more rapidly extracted solvent can be added to the quench extraction medium prior to addition of the emulsion. Thus, when the emulsion is added to the quench liquid, extraction of the more rapidly extracted solvent is retarded and more of the second, more slowly extracted solvent is removed. A method for encapsulating vitamins, food supplements, oil soluble substances at high loading (70 wt%) by the solvent o/w emulsion extraction technique is provided by Kvitnitsky et al.(78,79). Since evaporating the solvent from the dispersion is not applicable for delicate and sensitive compounds and it is not effective, because diffusion of solvent through a hard polymer wall is very slow, water at 10-30 times higher than the whole quantity of the organic solvent is added to the emulsion for extracting the solvent.

Dawson and Koppenhagen(80)employed a relatively high nonionic emulsifier concentration (5-15 wt%) in an emulsion-extraction method particularly applicable to those active agents that are susceptible to thermal degradation at temperatures above room temperature (i.e. 20 °C) such as enzymes, hormones and antigens. Eyles et al.(81) used the w/o/w and o/w/o emulsions to produce biodegradable microparticles that stimulate production of cytokines in a host cell, and contain single-stranded ribonucleic acid material, a stabilizing agent and a biologically active macromolecule where the outer surface of the microparticle is free from adsorbed molecules. Polysaccharides such as starch have been used as a matrix for encapsulation many active ingredients including proteins.

Wen and Anderson(82) prepared double wall microspheres using two biodegradable polymers by the o/w emulsification solvent extraction process. Futo et al.(83)used a relatively large molecular weight (11,000 to about 27,000) lactic acid polymer or its salt to produce microspheres with prolonged release over a long period of time with a suppressed initial excessive release of a watersoluble LHRH derivative via single or double emulsion.

Ducrey et al.(84)incorporated LHRH in the form of a water insoluble peptide salt (The LHRH agonist triptorelin pamoate) to provides slow release microparticles made of a copolymer of the PLGA type (at least 75 % of lactic acid) by the emulsion method.

A method of encapsulating DNA retaining its ability to induce expression of its coding sequence in a microparticle for oral administration prepared using the w/o/w emulsion and using biodegradable polymers under reduced shear is produced by Jones et al.(86–90). In addition, Little et al.(91)provided a high throughput method of preparing multiple (at least 10) different microparticle formulations (containing plasmid DNA) in parallel based on the double emulsion/solvent evaporation technique. The encapsulation of hormones such as calcitonin for the sustained release delivery has been achieved by Woo et al.(92). Biodegradable microspheres prepared using o/w emulsion technique and incorporating release-modifying agents and pH-stabilizing agents that resist changes in pH upon the addition of small amounts of acid or alkali such as basic amino acids, such as L-arginine were prepared(93). According to the disclosure of the invention, sustained release is affected by the unique interplay of the components of the novel microsphere delivery system.

Reslow et al.(94)utilized starch to encapsulate vaccines using emulsification method. In process, an immunologically active substance (vaccine) is suspended in an aqueous starch solution with an amylopectin content exceeding 85% by weight before being mixed with an aqueous solution of a polymer having the ability of forming a two phase aqueous system. The starch droplets containing the vaccine are allowed to gel as the starch has capacity to gel naturally.

Encapsulation of nucleotides and growth hormone using simple or double emulsification methods was achieved by Johnson et al.(95)respectively. Similar to synthetic polymers, such as poly (lactic acid) or polyorthoesters, proteins have also been used to form microparticles or microspheres for drug delivery. Most are cross-linked in solution using glutaraldehyde, or hardened at elevated temperatures(96). Unfortunately, there are problems with significant loss of biological activity of incorporated materials and lack of controlled size and *in vivo* degradation rates.

Suslick et al.(97) produced surface modified microparticles that possess a novel protein shell, and a surface coating. The protein shell might consist of cross-linked albumin or other proteins with functional moieties for cross-linking, while the surface coating comprises polyethylene glycol, a second protein or an antibody. Microparticles are prepared via emulsification followed by protein agglomeration and cross-linking(98). The surface coating may be covalently-bonded to the cross-linked protein shell or it may be electrostatically adsorbed to the cross-linked protein shell. The surface of the microparticles can be altered to vary the *in vivo* pharma-cokinetics and biodistribution.

## CONCLUSION

Microfabricated system offers potential advantages over conventional drug delivery systems. Microspheres and microcapsules are established as unique carrier systems for many pharmaceuticals and can be tailored to adhere to targeted tissue systems. Hence, micro-capsules and microspheres can be used not only for controlled release but also for targeted delivery of drugs to a specific site in the body. Although significant advances have been made in the field of microencapsulation, there are still many challenges ahead in this field. Of particular importance are the development of cheaper biopolymers for the microencapsulation technology and the development of universally acceptable evaluation methods especially for bioadhesive microspheres. Therefore, the development of safe and efficient particular systems will require, in the future, indepth investigations of both the biological and technological aspects of these systems.
